# Obstructive Sleep Apnea and Female Reproductive Endocrine Diseases: A Mendelian Randomization and Mediation Analysis

**DOI:** 10.1155/ije/3604933

**Published:** 2026-04-16

**Authors:** Zhe Wang, Jie Qin, Jia Wang, Ze Chen, Ziyu Qiao, Ming Yan, Dahai Wu

**Affiliations:** ^1^ Department of Otolaryngology, General Hospital of Northern Theater Command, Shenyang, 110016, Liaoning, China, syjqzyy.com; ^2^ Postgraduate Training Base of Dalian Medical University in the General Hospital of Northern Theater Command, Dalian, 116044, Liaoning, China; ^3^ Postgraduate Training Base of Jinzhou Medical University in the General Hospital of Northern Theater Command, Jinzhou, 121013, Liaoning, China

**Keywords:** body mass index, endometriosis, female infertility, Mendelian randomization, obstructive sleep apnea, polycystic ovary syndrome

## Abstract

**Objective:**

This study aimed to evaluate the potential causal association between obstructive sleep apnea (OSA) and the risk of female reproductive endocrine diseases (REDs), including polycystic ovary syndrome (PCOS), endometriosis (EMs), and female infertility (FI) using Mendelian randomization (MR) analysis.

**Methods:**

Genome‐wide association study (GWAS) summary statistics were obtained from the FinnGen consortium, GWAS catalog, UK Biobank, and GIANT consortium, with a focus on individuals of European ancestry. Single‐nucleotide polymorphisms (SNPs) correlated with OSA were investigated in relation to REDs (PCOS, EMs, and FI) using the inverse‐variance weighted (IVW) method. Sensitivity analyses were used to test the consistency of the results. Furthermore, a two‐step MR was performed to quantify the proportion of the effect of body mass index (BMI)–mediated OSA on REDs.

**Results:**

Our primary MR analysis revealed that genetically predicted OSA was positively linked to increased PCOS (odds ratio [OR] 1.341, 95% confidence interval [CI] 1.014–1.774, *p* = 0.039), while no significant causal relationships were observed between OSA and EMs, or FI (all *p* > 0.05). Mediation analysis showed that BMI mediated 18% of the association between OSA and PCOS (OR = 1.053, 95% CI = 1.015–1.105).

**Conclusion:**

This study suggested that early detection and management of OSA in women may be a new strategy to improve PCOS in the future. Meanwhile, it also emphasized the importance of adjusting BMI as a therapeutic strategy to mitigate the effects of OSA on PCOS.

## 1. Introduction

Female reproductive endocrine disorders (REDs), including polycystic ovary syndrome (PCOS), endometriosis (EMs), and female infertility (FI), represent a major clinical burden in women’s health, affecting hormonal regulation, metabolic homeostasis, and quality of life [1]. These disorders share common pathophysiologic features such as aberrant gonadotropin‐releasing hormone (GnRH) secretion, insulin resistance, and chronic inflammation. However, the specific pathophysiologic pathways that mediate their association with systemic metabolic and inflammatory sequelae remain unclear [1].

Obstructive sleep apnea (OSA), a chronic breathing disorder characterized by sleep fragmentation and intermittent hypoxia (IH), has emerged as a global health burden affecting nearly 1 billion individuals worldwide [2–4]. While its cardiometabolic consequences are well established, emerging evidence suggests OSA may exert under‐recognized endocrine‐disrupting effects through hypoxia‐triggered pathways, including oxidative stress, sympathetic overactivation, and systemic inflammation [5–7]. This raises critical questions about its potential role in female REDs, particularly given the pathophysiological parallels between OSA‐induced metabolic disturbances (aberrant GnRH secretion, insulin resistance) and hormonal dysregulation observed in PCOS, EMs, and FI [7, 8].

However, the potential association between different REDs and OSA seems to be different. Studies have shown that the prevalence of OSA reaches 40%–60% in PCOS cohorts versus 9%–28% in controls [9]. FI patients demonstrate 2‐fold higher OSA risk [10]. And the relationship between EMs and OSA has not yet been clearly studied [11]. However, critical limitations plague existing research. First, traditional observational studies are susceptible to confounding factors, and the inference of causality is limited and unreliable. Second, randomized controlled trials are difficult to realize due to various practical factors.

Mendelian randomization (MR) is a potential causal inference method that uses genetic variation as an instrumental variable to obtain the effect of exposure factors on outcomes from observational data [12]. MR can reduce the effects of non‐measurement errors or confounding factors while avoiding reverse causality through Mendelian inheritance laws [12]. Therefore, we aimed to determine whether OSA is causally related to REDs and assess the extent to which body mass index (BMI) mediates the effects of OSA on REDs through MR analysis.

## 2. Materials and Methods

### 2.1. Study Design

**FIGURE 1:**
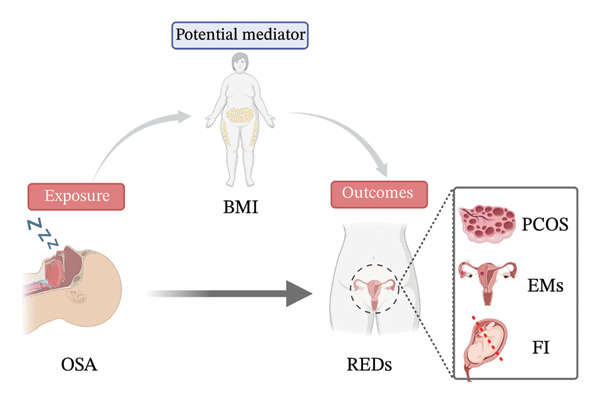
Framework for two‐sample MR in this study. The objective is to uncover the causal association between OSA and REDs, with potential mediation by BMI factors. Abbreviations: BMI, body mass index; EMs, endometriosis; FI, female infertility; OSA, obstructive sleep apnea; PCOS, polycystic ovary syndrome.

This study utilized publicly available data from previous genome‐wide association studies (GWAS). All data sources were approved by the institutional review committee in the respective studies. This study is reported following the Strengthening the Reporting of Observational Studies in Epidemiology Using Mendelian Randomization (STROBE‐MR) reporting guideline [13]. In this study, we explored the reciprocal causal relationship between OSA and REDs (PCOS, EMs, and FI) by two‐sample MR. Furthermore, we used two‐step MR to quantify the proportion of the effect of BMI‐mediated OSA on REDs. In our study, single‐nucleotide polymorphisms (SNPs) were defined as instrumental variables (IVs) (Figure 1) [14].

### 2.2. GWAS Summary Data Sources

All the datasets used are publicly available, and the participants in the GWAS were of European ancestry (Table 1). The GWAS summary‐level data for OSA were obtained from the FinnGen database (https://www.finngen.fi/en), which contains information from 54,698 patients and 399,035 controls [15]. The GWAS summary‐level data for PCOS, EMs, and FI were obtained from the GWAS catalog (https://www.ebi.ac.uk/gwas). Of these, the data accessing code for PCOS is GCST90044902, consisting of 3609 cases and 229,788 controls [16], with adjustments made for participant age to refine the study’s accuracy. The data accessing code for EMs is GCST90269970, consisting of 21,779 cases and 449,087 controls [17]. The data accessing code for FI is GCST90044466, consisting of 899 cases and 246,641 controls [18]. The GWAS summary‐level data for BMI were obtained from the UK Biobank and GIANT (Genetic Investigation of Anthropometric Traits) consortium (https://portals.broadinstitute.org/collaboration/giant/index.php/GIANT_consortium_data_files), which includes 434,794 female individuals [19]. All GWAS data are from different consortia or organizations; thus, there is no sample overlap.

**TABLE 1:** Summary of genome‐wide association studies (GWAS) datasets in our study.

	Phenotype	Source	Ethnicity	Participants	No. of SNPs	References
Exposure	OSA	FinnGen release 11	European	Cases: 54,698; controls: 399,035	21,306,794	Kurki et al. [15]

Outcomes	PCOS	GWAS catalog	European	Cases: 3609; controls: 229,788	14,073,390	Tyrmi et al. [16]
	EMs	GWAS catalog	European	Cases: 21,779; controls: 449,087	8,298,092	Rahmioglu et al. [17]
	FI	GWAS catalog	European	Cases: 899; controls: 246,641	11,832,029	Jiang et al. [18]

Covariate	BMI	UK Biobank and GIANT consortium	European	434,794 female individuals	2,473,355	Pulit et al. [19]

Abbreviations: BMI, body mass index; EMs, endometriosis; FI, female infertility; OSA, obstructive sleep apnea; PCOS, polycystic ovary syndrome; SNP, single‐nucleotide polymorphism.

### 2.3. IVs Selection and Data Harmonization

We included SNPs that were genome‐wide significant (*p* < 5 × 10^−8^). If there were no significant genome‐wide SNPs as IVs, SNPs with less than a genome‐wide significance level (*p* < 5 × 10^−6^) were used as candidate IVs. To ensure that IVs used for the OSA were independent, we excluded SNPs that had the linkage disequilibrium (LD) effect (*r* ^2^ < 0.001, clumping window = 10,000 kb). The 1000 Genomes European data were used as the reference panel [20]. We harmonized the exposure‐SNPs with outcome‐SNPs to exclude those being palindromic or incompatible [21]. Meanwhile, we rigorously filtered out those showing genome‐wide significant associations with the outcome, ensuring a robust selection of IVs. To assess the strength of the selected instrumental variables, we calculated the proportion of variance explained (*R* ^2^) for each SNP associated with OSA using the formula: *R* ^2^ = [2*β* ^2^ × EAF × (1 − EAF)]/[2*β* ^2^ × EAF × (1 − EAF) + 2SE^2^ × *N* × EAF × (1 − EAF)], where *β* is the estimated effect size, EAF is the effect allele frequency, SE is the standard error, and *N* is the sample size. The *F*‐statistic was calculated by the variance explained by SNPs for each exposure. This calculation was performed using the following formula: *F* = *R* ^2^ (*N* − *k* − 1)/*k* (1 − *R* ^2^), where *k* is the number of genetic variants and *N* is the sample size. To avoid bias from weak instruments, IVs with an *F*‐statistics < 10 were removed [22]. The selected instrumental SNPs for each analysis are listed in Table S1 (OSA‐REDs), Table S2 (PCOS‐OSA and EMs‐OSA), Table S3 (OSA‐BMI), and Table S4 (BMI‐PCOS).

### 2.4. Primary Analysis

We performed two‐sample MR to evaluate the causal relationship between OSA and PCOS, EMs, and FI, respectively. These results were designated as total effects. In addition, to exclude reverse causal effects on the mediating pathway, we also performed reverse MR analysis on the two samples.

### 2.5. Mediation Analysis

We further conducted a mediation analysis using a two‐step MR design to explore whether BMI mediates the causal pathway from OSA to REDs (PCOS, EMs, or FI) outcome. First, we estimated the causal effects of exposure on the mediator and the mediator on the outcome. In cases wherein there was evidence of exposure influencing the mediator, which subsequently impacted the risk of outcome, we utilized the “product of coefficients” method to assess the indirect effect of exposure on outcome risk through the mediator. Second, the total effect of exposure on outcome was decomposed into the direct effect of exposure on outcome and the indirect effect mediated through a mediator [23]. The percentage mediated effect was calculated by dividing the indirect effect by the total effect. Meanwhile, 95% confidence intervals (CIs) were calculated using the delta method.

### 2.6. Statistical Analysis

The multiplicative random‐effect inverse variance‐weighted (IVW) method is considered to have the highest ability to detect causality in MR analysis and is regarded to be the primary criterion for assessing the result. The weighted median and the MR‐Egger are used as supplementary methods [24]. The statistical significance threshold is usually set at 0.05. Cochran’s *Q* test is used to assess the heterogeneity among individual SNPs. If significant heterogeneity is observed, the random‐effects model is chosen [25]. The stability of the results is assessed using MR‐Egger regression and MR pleiotropy residual sum and outlier (MR‐PRESSO). MR‐Egger regression can detect directional pleiotropy, taking into account the possibility of nonzero intercepts [26]. MR‐PRESSO can identify and remove any horizontal pleiotropic outlier by calculating *p* values for each SNP [27]. Full results of heterogeneity and pleiotropy tests are provided in Table S5. The leave‐one‐out sensitivity method is used to assess whether the results are affected by the removal of any single SNP. Scatterplots and forest plots are generated to further examine the sensitivity of the results. All analyses were performed using the packages TwoSampleMR (version 0.6.6) and MR‐PRESSO (version 1.0) in R (version 4.4.1).

## 3. Results

### 3.1. Association of OSA With REDs (PCOS, EMs, and FI)

**FIGURE 2:**
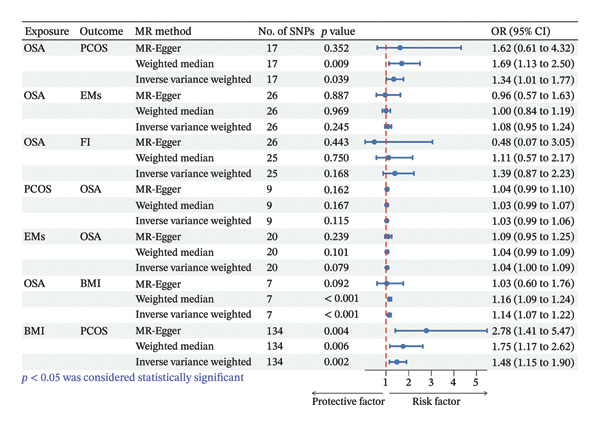
Forest plot to visualize the causal effects of BMI with OSA and REDs. Abbreviations: BMI, body mass index. OSA, obstructive sleep apnea; REDs, reproductive endocrine.

We conducted an MR analysis to evaluate the causal relationship between OSA and PCOS, EMs, and FI. A total of 1981 SNPs were extracted under the threshold of genome‐wide statistical significance (*p* < 5 × 10^−8^). After a series of quality control steps, a total of 17 independent SNPs were identified as IVs for PCOS, 26 independent SNPs were identified as IVs for EMs, and 25 independent SNPs were identified as IVs for FI. The mean *R* ^2^ for OSA‐associated SNPs was 9.50653 × 10^−5^, 9.0335 × 10^−5^, and 9.07858 × 10^−5^. There was no weak instrument bias, as the F‐statistics of IVs were all greater than 10. All selected instrumental SNPs and their corresponding *R* ^2^ and *F*‐statistics are listed (Table S1). The MR IVW analysis showed that a genetically predicted OSA was associated with a higher risk of PCOS (odds ratio [OR], 1.34; 95% CI, 1.01–1.77; *p* = 0.039) (Figure 2). There was no evidence that OSA is associated with EMs and FI (Figure 2).

The results of Cochran’s *Q* test, evaluated by the IVW test and MR‐Egger, showed no significant heterogeneity between the OSA and PCOS, EMs, and FI. There was no evidence of horizontal pleiotropy according to the results of the MR‐Egger regression analysis (Table S5). The findings were graphically depicted in scatterplots (Figures 3(a)–3(c)). In addition, MR‐PRESSO analysis did not find any significant outliers (Table S5). The leave‐one‐out results further validated data robustness (Figures 4(a)–4(c)).

FIGURE 3(a) Scatter plot for the causal association between OSA and PCOS; (b) scatter plot for the causal association between OSA and EMs; (c) scatter plot for the causal association between OSA and FI; (d) scatter plot for the causal association between PCOS and OSA; (e) scatter plot for the causal association between OSA and BMI; (f) scatter plot for the causal association between BMI and PCOS. Abbreviations: BMI, body mass index; EMs, endometriosis; FI, female infertility; MR, Mendelian randomization; OSA, obstructive sleep apnea; PCOS, polycystic ovary syndrome; SNP, single‐nucleotide polymorphism.

**Figure:**
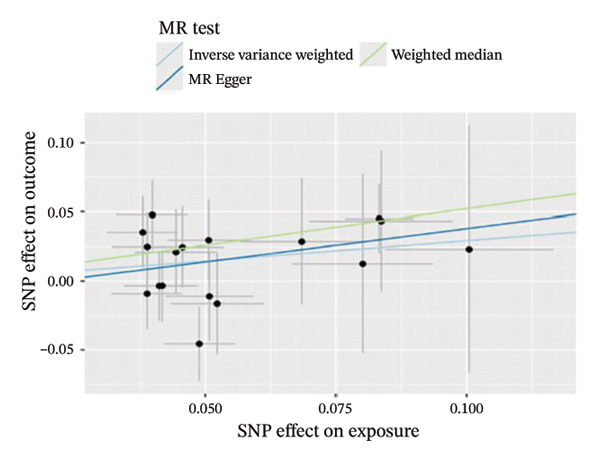
(a)

**Figure:**
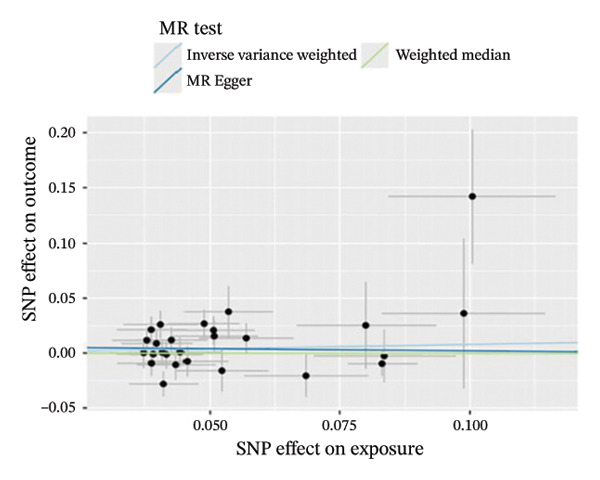
(b)

**Figure:**
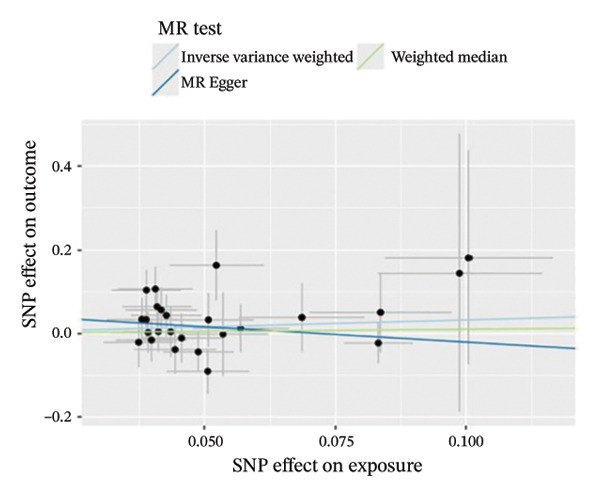
(c)

**Figure:**
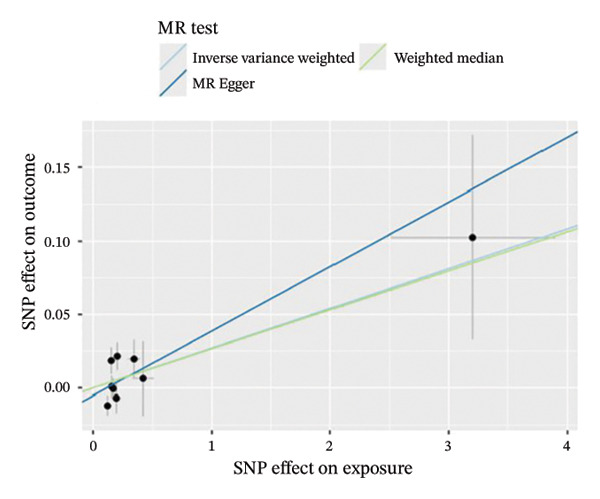
(d)

**Figure:**
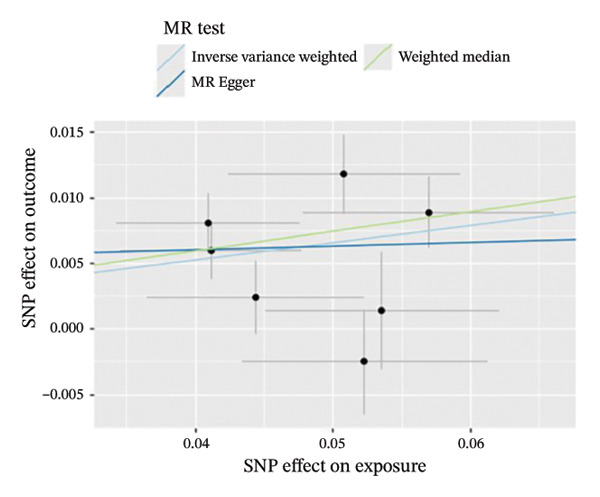
(e)

**Figure:**
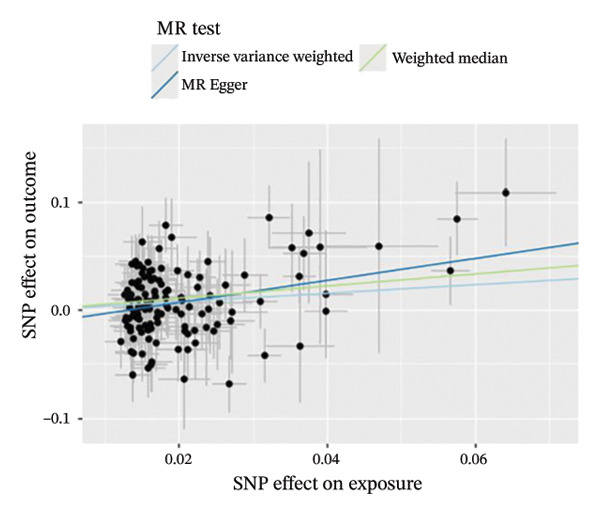
(f)

FIGURE 4(a) Leave‐one‐out regression analysis for the causal association between OSA and PCOS; (b) leave‐one‐out regression analysis for the causal association between OSA and EMs; (c) leave‐one‐out regression analysis for the causal association between OSA and FI; (d) leave‐one‐out regression analysis for the causal association between PCOS and OSA; (e) leave‐one‐out regression analysis for the causal association between OSA and BMI; (f) leave‐one‐out regression analysis for the causal association between BMI and PCOS. Abbreviations: BMI, body mass index; EMs, endometriosis; FI, female infertility; MR, Mendelian randomization; OSA, obstructive sleep apnea; PCOS, polycystic ovary syndrome.

**Figure:**
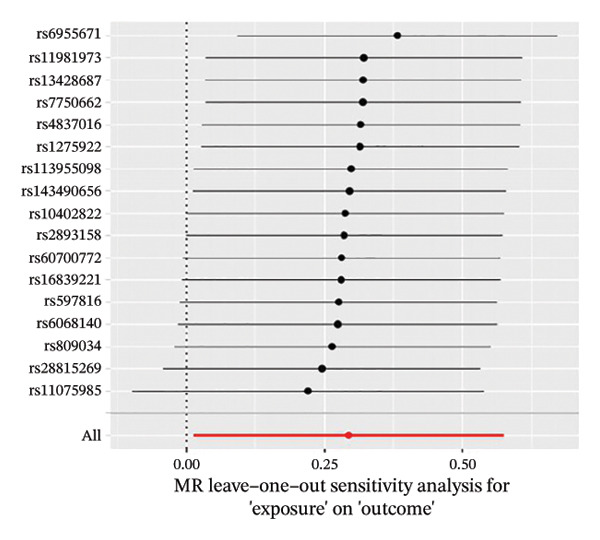
(a)

**Figure:**
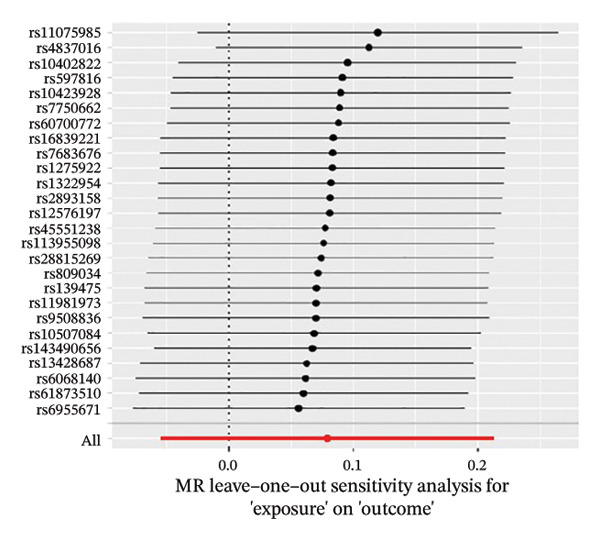
(b)

**Figure:**
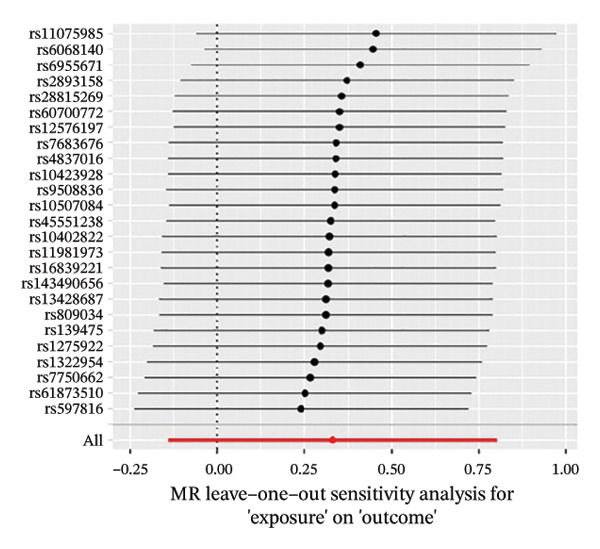
(c)

**Figure:**
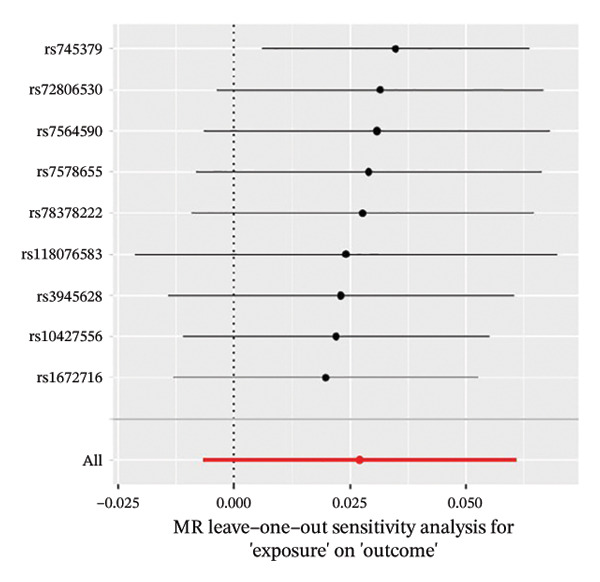
(d)

**Figure:**
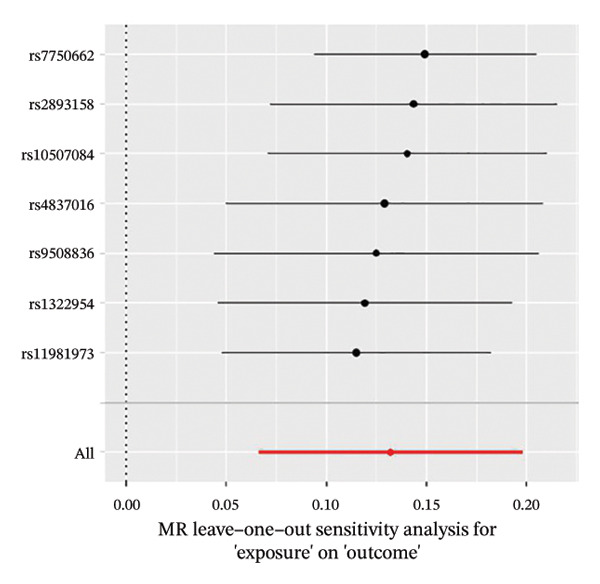
(e)

**Figure:**
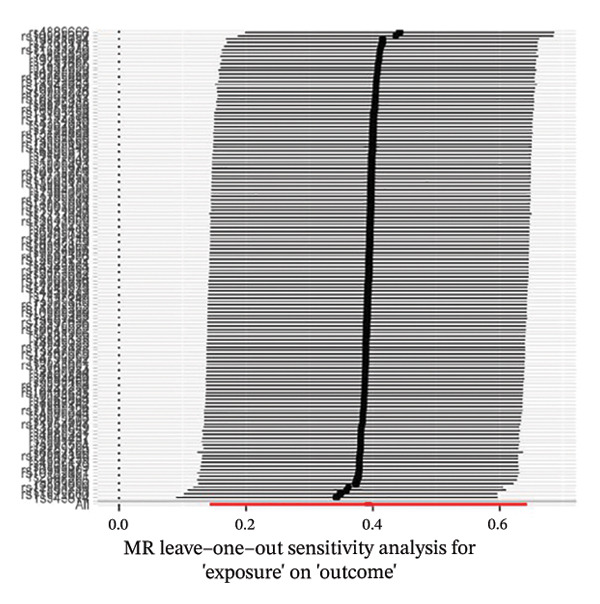
(f)

### 3.2. Reverse Causality Between REDs and OSA

We performed a reverse MR analysis to evaluate the causal relationship between PCOS and OSA. Since PCOS did not reach the level of gene‐wide significance for SNPs, SNPs with less than genome‐wide significance (*p* < 5 × 10^−6^) were used as instrumental variables. A total of 549 SNPs were extracted under the threshold of genome‐wide statistical significance. We extracted nine genome‐wide significant SNPs as instrumental variables after a series of quality control steps, and all F statistics of the genetic variants proxying sleep traits were greater than 10 (Table S2). The results of our MR analysis showed no reverse causality for genetically predicted PCOS on OSA. This result indicated that there was no causality for genetically predicted PCOS on OSA (OR, 1.08; 95% CI, 0.95–1.24; *p* = 0.245) (Figure 2).

The results of Cochran’s *Q* test showed no significant heterogeneity between the PCOS and OSA. The results of the MR‐Egger regression analysis found no evidence of horizontal pleiotropy (Table S5). The findings were graphically depicted in scatterplots (Figure 3(d)). In addition, the MR‐PRESSO test detected one outlier. After removing the outlier, the results remained robust (Table S5). The leave‐one‐out results further validated data robustness (Figure 4(d)).

We further examined the potential reverse causal effects of EMs and FI on OSA risk. For EMs, 20 independent genome‐wide significant SNPs (*p* < 5 × 10^−8^) were used as instrumental variables (Table S2). The IVW analysis showed no causal effect of genetic liability to EMs on OSA (OR: 1.04, 95% CI: 0.99–1.08; *p* = 0.08) (Figure 2). Although heterogeneity was noted, there was no evidence of horizontal pleiotropy. In addition, MR‐PRESSO analysis did not find any significant outliers (Table S5). For FI, due to the limited number of cases in the FI GWAS (*n* = 899), no robust instrumental variables could be identified, precluding a reliable reverse MR analysis.

### 3.3. Association of OSA With BMI

After a series of quality control steps, seven independent SNPs were identified as IVs for BMI. The F‐statistics of IVs were all greater than 10. All selected instrumental SNPs are listed (Table S3). The MR IVW analysis showed that a genetically predicted OSA was associated with a higher risk of BMI (OR, 1.14; 95% CI, 1.07–1.22; *p* < 0.001) (Figure 2).

Although heterogeneity was noted, there was no evidence of horizontal pleiotropy (Table S5). The findings were graphically depicted in scatterplots (Figure 3(e)). In addition, the MR‐PRESSO test detected five outliers. After removing the outliers, the associations remained robust (Table S5). The leave‐one‐out results further validated data robustness (Figure 4(e)).

### 3.4. Association of BMI With PCOS

We conducted an MR analysis to evaluate the causal relationship between BMI and PCOS. A total of 14,723 SNPs were extracted under the threshold of genome‐wide statistical significance (*p* < 5 × 10^−8^). After a series of quality control steps, a total of 147 independent SNPs were identified as IVs for PCOS. There was no weak instrument bias, as the *F*‐statistics of IVs were all greater than 10. All selected instrumental SNPs are listed (Table S4). The MR IVW analysis showed that a genetically predicted BMI was associated with a higher risk of PCOS (OR, 1.48; 95% CI, 1.15–1.90; *p* = 0.002) (Figure 2).

The results of Cochran’s *Q* test, evaluated by the IVW test and MR‐Egger, showed no significant heterogeneity between the PCOS and BMI. There was no evidence of horizontal pleiotropy according to the results of the MR‐Egger regression analysis (Table S5). The findings were graphically depicted in scatterplots (Figure 3(f)). In addition, MR‐PRESSO analysis did not find any significant outliers (Table S5). The leave‐one‐out results further validated data robustness (Figure 4(f)).

Notably, the MR‐Egger method yielded wider CIs compared to the IVW and weighted median methods for several analyses. This is an expected consequence of the MR‐Egger approach, which provides consistent estimates under the InSIDE assumption but with reduced statistical power due to the estimation of an intercept term to account for directional pleiotropy [26]. The wider intervals reflect this trade‐off between robustness to pleiotropy and precision and do not indicate instability of the results. Importantly, the direction of effect estimates remained broadly consistent across methods, supporting the robustness of our primary findings.

### 3.5. Proportion of the Association Between OSA and PCOS Mediated by BMI

We analyzed BMI as a mediator of the pathway from OSA to PCOS. We found that OSA was associated with increased BMI, which in turn was associated with an increased risk of PCOS. Our study showed that BMI accounted for 17.7% of the increased risk of OSA associated with PCOS (OR = 1.053, 95% CI = 1.015–1.105) (see Table 2).

**TABLE 2:** Proportion of the association between OSA and PCOS mediated by BMI.

Parameter	Effect (*β*)	SE	OR	CI 95%
Total effect (OSA‐PCOS)	0.294	0.143	1.341	1.014–1.774
Indirect effect (OSA‐BMI‐PCOS)	0.052	0.021	1.053	1.012–1.097
Direct effect (OSA‐PCOS)	0.242	0.144	1.273	0.961–1.689

Abbreviations: BMI, body mass index; OSA, obstructive sleep apnea; PCOS, polycystic ovary syndrome.

## 4. Discussion

In this MR study, we systematically assessed the associations of genetically predicted OSA with the risk of REDs, including PCOS, EMs, and FI. Our findings support a potential causal relationship between OSA and the risk of PCOS. In contrast, we did not identify any statistically significant associations between the genetically predicted OSA and the risk of EMs or FI. However, these invalid results should be interpreted with caution. Furthermore, our mediation analysis showed that BMI mediated approximately 17.7% of the association between OSA and PCOS.

The female reproductive endocrine system is the core of maintaining women’s reproductive health and fertility functions, and its normal functioning directly affects the menstrual cycle, ovulation, conception, and maintenance of pregnancy. With the development of society and changes in lifestyle, the incidence of REDs is gradually increasing. These diseases not only affect women’s fertility but also have long‐term effects on their health. Therein, PCOS is one of the most prevalent endocrine and metabolic disorders in reproductive‐aged females, including obesity, insulin resistance, hyperinsulinemia, and dyslipidemia [28, 29]. EMs is a disease characterized by endometrial tissue outside the uterus, which affects 10% of reproductive‐aged females worldwide and leads to chronic painful symptoms and infertility in severe cases [28, 30]. FI is defined as the failure to conceive after 12 months of regular unprotected sexual intercourse. It has been reported that FI has become a major public health problem affecting 8%–12% of reproductive‐aged couples [28, 31]. Because these diseases place a serious health, economic, and social burden on women, it is particularly important to understand their underlying pathogenesis [28].

OSA is a condition characterized by episodes of complete or partial obstruction of the upper airway during sleep, resulting in disturbances in the structure of nocturnal sleep and frequent microarousals. Studies have shown a correlation between OSA and REDs. Sleep fragmentation or sleep deprivation due to OSA may affect a woman’s hormone levels [32], which in turn interfere with the menstrual cycle, ovulation function, and hormone production. For example, OSA can lead to imbalances in hormones such as melatonin and cortisol, which play an important role in regulating female reproductive health. In addition, chronic sleep problems may exacerbate the symptoms of REDs and affect insulin sensitivity and weight management, further increasing the risk of the disease [7]. On the other hand, erratic levels of hormones may also exacerbate emotional problems such as anxiety and depression, creating a vicious cycle between OSA and RED [33]. Therefore, effective treatment of OSA may help alleviate the symptoms of REDs in women and promote overall health.

Previous studies have explored the relationship between OSA and PCOS, while the relationship between OSA and EMs and FI was less well studied. A meta‐analysis based on clinical studies reported a higher risk of OSA in adult women with PCOS compared with non‐PCOS patients, but no difference in adolescent patients [34]. In another meta‐analysis, Kahal et al. reported that 35.0% of women with PCOS coexisted with OSA, and the proportion of obese PCOS women with OSA (33.33%–40.91%) was significantly higher than lean PCOS women (0%) [35]. Meanwhile, several observational epidemiologic studies have reported that OSA can exacerbate the clinical manifestations of PCOS [36, 37]. Conversely, women with PCOS are at increased risk of developing OSA compared to control women, irrespective of obesity [38]. Similarly, Lim et al. found that OSA is more common in FI and can increase the chances of FI. Therefore, when suffering from FI, patients should be screened for signs and symptoms of OSA, which may help improve female fertility [11]. However, there are relatively few studies on the independent correlation between EMs and OSA. Boneva et al. found an association between EMs and OSA only in combination with chronic fatigue syndrome [10]. However, these studies were observational studies, and their results may have been influenced by reverse causality or other potential confounders. These limitations make it difficult to determine whether they are purely correlated or causally related.

This study supported a potential causal relationship between OSA and PCOS risk, which is consistent with previous observational studies and meta‐analyses. The association between OSA and PCOS may be mediated through a variety of mechanisms, including insulin resistance, hormonal dysregulation, and chronic inflammation. In OSA patients, IH may exacerbate insulin sensitivity, leading to elevated insulin levels. Hyperinsulinemia increases ovarian androgen production, thereby exacerbating the symptoms of PCOS. Second, the stress response triggered by sleep disorders causes OSA patients to affect hormone regulation by increasing cortisol levels. Elevated levels of cortisol further contribute to insulin resistance and may affect ovarian function, leading to a hyperandrogenic state in PCOS patients [9, 39]. In addition, chronic inflammation is a common feature of OSA and PCOS. Studies have shown that elevated levels of inflammatory markers such as CRP and TNF‐α are observed in both OSA and PCOS patients. OSA induces systemic inflammation through repeated episodes of hypoxia and reoxygenation, and PCOS is associated with elevated inflammatory cytokines. This common inflammatory pathway may exacerbate both OSA and PCOS conditions, leading to worse outcomes.

Unlike PCOS, this study did not find a significant genetic predictive correlation between OSA and EMs or FI. However, these results should be interpreted with caution. EMs is primarily an estrogen‐dependent disease characterized by the proliferation of ectopic endometrial tissue [40]. The pathogenesis of this disease primarily centers on local inflammation, progesterone resistance, and altered cellular metabolism [41–43]. These mechanisms show little direct overlap with the systemic effects of IH and sleep fragmentation associated with OSA. Interestingly, a study has shown that hypoxia‐inducible factor 1α (HIF‐1α)—a central mediator of cellular responses to hypoxia in OSA—has been shown to enhance the survival of endometrial stromal cells through metabolic adaptation [44]. This suggests that hypoxia induced by OSA may actually improve existing lesions rather than trigger them. This could potentially explain the absence of clear genetic associations in our analysis. Additionally, while Yang et al.’s analysis identified a causal association between insomnia and the risk of EMs, no significant associations were found for other sleep characteristics such as circadian rhythm type, sleep duration, daytime napping, or daytime sleepiness [45]. This provides important contextual information, suggesting that the lack of association between OSA and EMs may be consistent with the specificity of sleep‐related effects. For FI, several factors may explain the absence of a detectable genetic signal. First, statistical power was limited. Post hoc calculations indicated that our analysis had only about 30%–40% power to detect the observed effect for FI. This low power is largely attributable to the small number of FI cases (*n* = 899), underscoring the need for cautious interpretation. Second, although OSA could theoretically impact fertility through mechanisms such as oxidative damage to oocytes [46], human oocyte reserves possess substantial physiological redundancy, and it remains uncertain whether OSA‐mediated pathways alone are sufficient to reach the threshold required to impair fertility [47, 48]. Animal models suggest that substantial follicle loss (≥ 50%) is necessary to affect fertility, a level unlikely to be attributable to OSA‐related mechanisms alone [48]. Third, the FI phenotype is highly heterogeneous, encompassing diverse etiologies including tubal factors, ovulatory disorders, uterine abnormalities, and unexplained causes. This heterogeneity in the outcome GWAS may dilute any specific effect of OSA, making it more difficult to detect in a genetic framework. Therefore, while our findings do not support a strong causal effect of OSA on FI, they cannot definitively rule out a modest association given the limited statistical power. Further studies with larger, more phenotypically refined FI cohorts are warranted to clarify whether any relationship exists. BMI plays an important role in mediating the correlation between OSA and PCOS. Based on overlapping hormonal and metabolic factors, the correlation between BMI and OSA and PCOS has become an important area of research. On the one hand, OSA can lead to metabolic dysfunction, causing increased appetite and insulin resistance. Insulin resistance can alter fat distribution and promote weight gain, creating a vicious cycle that exacerbates obesity and OSA. In addition, disturbances of sleep structure can affect the increased secretion of leptin and gastric hunger hormone, which ultimately leads to overeating and weight gain [49]. At the same time, patients with OSA may experience changes in the distribution of body fluids, especially an increase in extracellular fluid during sleep. This fluid retention can lead to transient weight gain in patients with OSA [49]. On the other hand, BMI plays an important role in the presentation and management of PCOS, which seriously affects women’s metabolic and reproductive health [50]. Insulin resistance is exacerbated by increased inflammation and altered hormone levels due to excess body fat, especially abdominal visceral fat [51]. Insulin resistance can further disrupt normal ovarian function, leading to anovulation or reduced ovulation, which is a classic sign of PCOS [52]. These symptoms can affect female fertility and lead to infertility. Therefore, BMI may contribute to the development of PCOS by mediating insulin resistance and hormone level disruption in OSA patients. This study found that BMI mediated about 17.7% correlation between OSA and PCOS, suggesting that obesity may be a key bridge between OSA and PCOS. This finding provides new ideas for the management of comorbidities in OSA and PCOS, suggesting that weight management and metabolic health may help alleviate clinical symptoms in patients with OSA and PCOS. Meanwhile, although this study identified BMI as an important mediating factor, it is worth noting that other pathophysiological pathways may also contribute to the association between OSA and PCOS. Insulin resistance is a common feature of both diseases and a potent underlying mediator. IH induced by OSA exacerbates systemic insulin resistance and promotes hyperinsulinemia, thereby stimulating ovarian androgen secretion. This is precisely the core characteristic of PCOS [9, 30]. Additionally, chronic low‐grade inflammation is a shared characteristic of OSA and PCOS [53, 54]. OSA‐induced IH and fragmented sleep can elevate levels of proinflammatory cytokines (such as TNF‐α and IL‐6) and CRP, which may exacerbate insulin resistance and directly interfere with follicular development and hormonal regulation [5, 7]. Future studies should conduct GWAS with sufficient statistical power to quantitatively analyze these intermediate molecular characteristics (such as triglyceride‐glucose index, HOMA‐IR [55], and specific inflammatory markers) in order to formally evaluate their mediating role in the causal chain linking OSA to PCOS.

Additionally, while our sensitivity analyses did not reveal statistically significant horizontal pleiotropy, understanding the potential biological connections between the genetic instruments for OSA and the pathophysiology of PCOS is valuable. For example, rs11075985, a recognized obesity susceptibility locus located in the vicinity of the FTO gene, is strongly associated with sleep apnea, female waist‐to‐hip ratio, and high‐density lipoprotein cholesterol levels. This links genetic susceptibility to OSA with obesity and metabolic disorders. The rs11981973 variant in AUTS2 is associated with BMI and metabolic syndrome in European and East Asian populations, further supporting shared metabolic pathways. Located in the vicinity of GIPR, rs10423928 is a gene involved in incretin signaling and insulin secretion, and is strongly associated with multiple metabolic traits including BMI, waist circumference, visceral adipose tissue, obesity, blood pressure, and glucose metabolism. These associations directly implicate insulin resistance mechanisms central to PCOS.

Beyond the individual associations of specific SNPs, an integrative examination of their collective biological functions reveals a convergent mechanistic theme. Among these, FTO, GIPR, and AUTS2 are all concentrated in pathways closely associated with energy balance, glucose metabolism, and adipocyte function. The FTO gene is a key regulator of energy balance and fat production, and its variations influence body composition, fat distribution, and appetite regulation [56, 57]. The GIPR gene encodes the receptor for glucose‐dependent insulinotropic polypeptide, a key incretin hormone that potentiates insulin secretion, directly implicating pancreatic β‐cell function [58]. The AUTS2 gene was initially identified in the field of neurodevelopment and has since been shown to function as a pleiotropic locus associated with metabolic traits [59, 60]. Against this backdrop, the function of this gene may reflect shared genetic pathways between central nervous system function and peripheral metabolic regulation. The convergence of these genetic loci on pathways involving insulin resistance, fat accumulation, and metabolic dysfunction supports the model that genetic susceptibility to OSA primarily increases PCOS risk through metabolic dysfunction pathways. This pattern is consistent with vertical pleiotropy, where genetic variants affect the outcome via the exposure. The absence of detectable horizontal pleiotropy in our statistical tests further supports the validity of our causal inference.

Although this study provides new evidence for the relationship between OSA and REDs, it remains critical to carefully interpret the findings while considering the limitations specific to this study and MR in general. First, our study is based exclusively on individuals of European ancestry, which may limit the generalizability of our findings to other ethnic populations. Genetic architecture, lifestyle factors, and prevalence of both OSA and REDs can vary across ethnic groups, potentially influencing the observed causal relationships. Future MR studies utilizing multi‐ethnic GWAS data are warranted to validate and extend our conclusions to diverse populations. Second, OSA’s GWAS data include both males and females, whereas REDs’ GWAS data are only for females, which may have some impact on the accuracy of the results. However, due to the lack of demographic data in the original study, we were unable to perform subgroup analyses stratified by gender to avoid this bias [28]. Genetic evidence suggests a significant shared genetic basis for OSA across sexes, with many risk loci demonstrating consistent effects in men and women [61]. Furthermore, our sensitivity analyses, which found no evidence of horizontal pleiotropy, support the robustness of our findings against major violations of MR assumptions that might arise from sex differences in genetic architecture. Nonetheless, future GWAS studies on OSA that incorporate gender stratification will help validate our findings. Third, because OSA is a binary exposure, possible selection bias due to underdiagnosis cannot be accurately assessed. Fourth, the lack of stratified data on OSA severity limits in‐depth exploration of the relationship between OSA severity and REDs. Fifth, the null association between OSA and FI must be interpreted with caution due to limited statistical power. The FI GWAS included only 899 cases. Post hoc calculations indicate that, based on the observed effect size (OR = 1.39) and assuming the genetic instrumental variable accounts for approximately 2% of the variance in OSA, our study had only about 30%–40% power to detect such an effect at *α* = 0.05. Therefore, although the results do not support a significant effect, caution is warranted in interpreting these findings. Future validation through larger‐scale FI GWAS studies is required. Finally, although our study ruled out the interference of horizontal pleiotropy by the MR‐Egger intercept and MR‐PRESSO methods, it still cannot be completely ruled out whether exposure‐related SNPs have an impact on the risk of REDs in other ways.

## 5. Conclusion

This MR study supported the hypothesis that OSA may be associated with the development of REDs in women. We found a causal correlation between OSA and PCOS, and that BMI plays a mediating role in this association. Meanwhile, we did not observe causal correlations between OSA and EMs and FI. However, these findings require further confirmation. Our results suggested that early detection and management of OSA in women may be a new strategy to improve PCOS in the future. Meanwhile, it also emphasized the importance of adjusting BMI as a therapeutic strategy to mitigate the effects of OSA on PCOS.

## Funding

This work was supported by the Joint Project of Liaoning Provincial Science and Technology Program (Key R&D Program) (Grant 2023JH2/101800014).

## Conflicts of Interest

The authors declare no conflicts of interest.

## Supporting Information

TABLE S1: Characteristics of significant SNPs with genome‐wide associations (*p* < 5 × 10^−8^) for OSA on REDs.

TABLE S2: Characteristics of significant SNPs with genome‐wide associations (*p* < 5 × 10^−8^ or *p* < 5 × 10^−6^) for PCOS and EMs on OSA.

TABLE S3: Characteristics of significant SNPs with genome‐wide associations (*p* < 5 × 10^−8^) for OSA on BMI.

TABLE S4: Characteristics of significant SNPs with genome‐wide associations (*p* < 5 × 10^−8^) for BMI on PCOS.

TABLE S5: Heterogeneity and pleiotropy in MR analyses.

## Supporting information


**Supporting Information** Additional supporting information can be found online in the Supporting Information section.

## Data Availability

Data are available in the Supporting Information.
