# Supplementary material for: Obstructive Sleep Apnea and Female Reproductive Endocrine Diseases: A Mendelian Randomization and Mediation Analysis
Source: Int J Endocrinol. 2026 Apr 16;2026:3604933. doi: 10.1155/ije/3604933 (PMC13087510; doi:10.1155/ije/3604933)
Supplement: Supplementary file 1 — Supporting Information Additional supporting information can be found online in the Supporting Information section. [file IJE-2026-3604933-s001.zip › STROBE-MR checklist.docx]

**STROBE-MR checklist of recommended items to address in reports of Mendelian randomization studies**

| **Item No.** | **Section** | **Checklist item** | **Page No.** | **Relevant text from manuscript** |
| --- | --- | --- | --- | --- |
| 1 | **TITLE and ABSTRACT** | Indicate Mendelian randomization (MR) as the study’s design in the title and/or the abstract if that is a main purpose of the study | 1-2 | Title: "Obstructive sleep apnea and female reproductive endocrine disorder: a Mendelian randomization and mediation analysis"  Abstract: "using Mendelian randomization (MR) analysis |
|  | **INTRODUCTION** |  |  |  |
| 2 | **Background** | Explain the scientific background and rationale for the reported study. What is the exposure? Is a potential causal relationship between exposure and outcome plausible? Justify why MR is a helpful method to address the study question | 2-3 | Exposure: OSA  Outcome: REDs (PCOS, EMs, FI)  Rationale: OSA may disrupt endocrine pathways via hypoxia (Oxidative stress, sympathetic activation). Pathophysiological parallels exist between OSA-induced metabolic disturbances (e.g., insulin resistance) and REDs. Observational studies cannot resolve reverse causality (e.g., obesity confounding). MR avoids this by using genetic instruments. |
| 3 | **Objectives** | State specific objectives clearly, including pre-specified causal hypotheses (if any). State that MR is a method that, under specific assumptions, intends to estimate causal effects | 3 | "Purpose: Evaluate potential causal association between OSA and REDs (PCOS, EMs, FI) using MR. Quantify BMI-mediated proportion via two-step MR." |
|  | **METHODS** |  |  |  |
| 4 | **Study design and data sources** | Present key elements of the study design early in the article. Consider including a table listing sources of data for all phases of the study. For each data source contributing to the analysis, describe the following: |  |  |
|  | a) | Setting: Describe the study design and the underlying population, if possible. Describe the setting, locations, and relevant dates, including periods of recruitment, exposure, follow-up, and data collection, when available. | 3-4 | Design: Two-sample MR + mediation analysis  Data Sources: Public GWAS summary statistics (FinnGen, GWAS Catalog, UK Biobank/GIANT) for European ancestry (Table 1). |
|  | b) | Participants: Give the eligibility criteria, and the sources and methods of selection of participants. Report the sample size, and whether any power or sample size calculations were carried out prior to the main analysis | 4 | Eligibility: European ancestry. Sample sizes:  - OSA: 54,698 cases / 399,035 controls  - PCOS: 3,609 cases / 229,788 controls  - EMs: 21,779 cases / 449,087 controls  - FI: 899 cases / 246,641 controls  - BMI: 434,794 females. No power calculation mentioned. |
|  | c) | Describe measurement, quality control and selection of genetic variants | 4 | IV criteria: 1. Genome-wide significance (P<5×10^−8^; if none, P<5×10^−6^) 2. LD clumping (r^2^<0.001*r*^2^<0.001, window=10,000 kb) 3. F-statistic >10 (weak instrument exclusion) 4. Palindromic/ambiguous SNPs excluded. |
|  | d) | For each exposure, outcome, and other relevant variables, describe methods of assessment and diagnostic criteria for diseases | 4 | OSA: FinnGen database (diagnosis codes)  PCOS/EMs/FI: GWAS Catalog (disease codes)  BMI: UK Biobank/GIANT (anthropometric measures). |
|  | e) | Provide details of ethics committee approval and participant informed consent, if relevant | 4 | "All data sources were approved by institutional review committees in respective studies." |
| 5 | **Assumptions** | Explicitly state the three core IV assumptions for the main analysis (relevance, independence and exclusion restriction) as well assumptions for any additional or sensitivity analysis | 5 | Relevance: SNPs strongly associated with OSA (P<5×10^−8^) Independence: SNPs independent of confounders (LD clumping) Exclusion restriction: SNPs affect outcome only via exposure (tested via MR-Egger/MR-PRESSO for pleiotropy). |
| 6 | **Statistical methods: main analysis** | Describe statistical methods and statistics used |  |  |
|  | a) | Describe how quantitative variables were handled in the analyses (i.e., scale, units, model) | 5 | Scale: Binary exposures/outcomes-ORs  Model: Multiplicative random-effects IVW (primary), weighted median, MR-Egger. |
|  | b) | Describe how genetic variants were handled in the analyses and, if applicable, how their weights were selected | 5 | Harmonization to ensure allele alignment; ambiguous SNPs excluded. |
|  | c) | Describe the MR estimator (e.g. two-stage least squares, Wald ratio) and related statistics. Detail the included covariates and, in case of two-sample MR, whether the same covariate set was used for adjustment in the two samples | 5 | Primary: IVW  Mediation: Two-step MR ("product of coefficients")  Covariates: Age-adjusted (PCOS dataset). No overlap in covariate sets between samples. |
|  | d) | Explain how missing data were addressed | - | Not explicitly mentioned. |
|  | e) | If applicable, indicate how multiple testing was addressed | 5 | Significance threshold: P<0.05 (no correction stated). |
| 7 | **Assessment of assumptions** | Describe any methods or prior knowledge used to assess the assumptions or justify their validity | 5 | Pleiotropy: MR-Egger intercept + MR-PRESSO  Heterogeneity: Cochran’s Q test → random-effects if present. |
| 8 | **Sensitivity analyses and additional analyses** | Describe any sensitivity analyses or additional analyses performed (e.g. comparison of effect estimates from different approaches, independent replication, bias analytic techniques, validation of instruments, simulations) | 5 | Performed:  Weighted median, MR-Egger  MR-PRESSO (outlier removal)  Leave-one-out analysis  Reverse MR (for PCOS→OSA) |
| 9 | **Software and pre-registration** |  |  |  |
|  | a) | Name statistical software and package(s), including version and settings used | 5 | R (v4.4.1), TwoSampleMR (v0.6.6), MR-PRESSO (v1.0) |
|  | b) | State whether the study protocol and details were pre-registered (as well as when and where) | - | Not mentioned |
|  | **RESULTS** |  |  |  |
| 10 | **Descriptive data** |  |  |  |
|  | a) | Report the numbers of individuals at each stage of included studies and reasons for exclusion. Consider use of a flow diagram | - | No flow diagram; sample sizes in Table 1. |
|  | b) | Report summary statistics for phenotypic exposure(s), outcome(s), and other relevant variables (e.g. means, SDs, proportions) | 6-7 | **Key estimates:** OSA → PCOS: OR=1.34 (95% CI:1.01–1.77, P=0.039) BMI → PCOS: OR=1.48 (95% CI:1.15–1.90, P=0.002) Mediation: BMI mediated 17.7% (β=0.052, 95% CI:0.015–0.100) |
|  | c) | If the data sources include meta-analyses of previous studies, provide the assessments of heterogeneity across these studies | - | N/A (no meta-analysis). |
|  | d) | For two-sample MR:  i.  Provide justification of the similarity of the genetic variant-exposure associations between the exposure and outcome samples  ii.  Provide information on the number of individuals who overlap between the exposure and outcome studies | 4 | "All GWAS data are from different consortia → no sample overlap." |
| 11 | **Main results** |  |  |  |
|  | a) | Report the associations between genetic variant and exposure, and between genetic variant and outcome, preferably on an interpretable scale | 6-7 | IV strength: F-statistics >10 (S1–S4 Tables). |
|  | b) | Report MR estimates of the relationship between exposure and outcome, and the measures of uncertainty from the MR analysis, on an interpretable scale, such as odds ratio or relative risk per SD difference | 6-7 | **Main results:** OSA → PCOS: OR=1.34 (IVW) OSA → EMs/FI: Null (P>0.05) Mediation: Proportion=17.7% (Fig. 2, Text) |
|  | c) | If relevant, consider translating estimates of relative risk into absolute risk for a meaningful time period | - | Not calculated. |
|  | d) | Consider plots to visualize results (e.g. forest plot, scatterplot of associations between genetic variants and outcome versus between genetic variants and exposure) | 6-7 | Scatter plots (Fig. 3), Forest plots (Supplementary), Leave-one-out plots (Fig. 4). |
| 12 | **Assessment of assumptions** |  |  |  |
|  | a) | Report the assessment of the validity of the assumptions | 6-7 | **Pleiotropy:** MR-Egger intercept P>0.05 (Supplementary Table S5) **Heterogeneity:** Cochran’s Q P>0.05 (except BMI, handled via random-effects). |
|  | b) | Report any additional statistics (e.g., assessments of heterogeneity across genetic variants, such as *I^2^*, Q statistic or E-value) | 6-7 | MR-PRESSO: Outliers removed for OSA→BMI (5 SNPs) and PCOS→OSA (1 SNP). |
| 13 | **Sensitivity analyses and additional analyses** |  |  |  |
|  | a) | Report any sensitivity analyses to assess the robustness of the main results to violations of the assumptions | 6-7 | Consistent results across IVW, weighted median, MR-Egger (Supplementary Table S5). |
|  | b) | Report results from other sensitivity analyses or additional analyses | 6-7 | **Reverse MR:** PCOS → OSA: Null (OR=1.08, P=0.245) **Mediation:** Two-step MR for BMI. |
|  | c) | Report any assessment of direction of causal relationship (e.g., bidirectional MR) | 6 | Bidirectional MR confirmed OSA→PCOS (not reverse). |
|  | d) | When relevant, report and compare with estimates from non-MR analyses | 8 | Compared with observational studies. |
|  | e) | Consider additional plots to visualize results (e.g., leave-one-out analyses) | 6-7 | Leave-one-out plots (Fig. 4). |
|  | **DISCUSSION** |  |  |  |
| 14 | **Key results** | Summarize key results with reference to study objectives | 8 | "OSA causally linked to PCOS (OR=1.34), but not EMs/FI. BMI mediated 17.7% of OSA→PCOS effect." |
| 15 | **Limitations** | Discuss limitations of the study, taking into account the validity of the IV assumptions, other sources of potential bias, and imprecision. Discuss both direction and magnitude of any potential bias and any efforts to address them | 11 | 1. European-only ancestry  2. OSA GWAS includes males (REDs: females only)  3. Binary OSA exposure → underdiagnosis bias  4. Residual pleiotropy possible |
| 16 | **Interpretation** |  |  |  |
|  | a) | Meaning: Give a cautious overall interpretation of results in the context of their limitations and in comparison with other studies | 9-10 | Results align with observational studies (OSA-PCOS link) but clarify causality. Null for EMs/FI explained by pathophysiological differences. |
|  | b) | Mechanism: Discuss underlying biological mechanisms that could drive a potential causal relationship between the investigated exposure and the outcome, and whether the gene-environment equivalence assumption is reasonable. Use causal language carefully, clarifying that IV estimates may provide causal effects only under certain assumptions | 9-11 | OSA→PCOS: Hypoxia → insulin resistance → hyperandrogenism; inflammation (CRP/TNF-α).  BMI mediation: OSA → metabolic dysfunction → weight gain → PCOS. |
|  | c) | Clinical relevance: Discuss whether the results have clinical or public policy relevance, and to what extent they inform effect sizes of possible interventions | 10-11 | "Early OSA detection may improve PCOS management. Weight loss (BMI reduction) could mitigate OSA effects on PCOS." |
| 17 | **Generalizability** | Discuss the generalizability of the study results (a) to other populations, (b) across other exposure periods/timings, and (c) across other levels of exposure | 11 | Population: Limited to Europeans  Exposure: Binary OSA (severity not assessed)  Timing: Lifelong genetic effects. |
|  | **OTHER INFORMATION** |  |  |  |
| 18 | **Funding** | Describe sources of funding and the role of funders in the present study and, if applicable, sources of funding for the databases and original study or studies on which the present study is based | - | This work was supported by the project of Science and Technology Plan of Liaoning Province under Grant 2023JH2/101800014. |
| 19 | **Data and data sharing** | Provide the data used to perform all analyses or report where and how the data can be accessed, and reference these sources in the article. Provide the statistical code needed to reproduce the results in the article, or report whether the code is publicly accessible and if so, where | 12 | "All relevant data are within the manuscript and its Supporting Information files." |
| 20 | **Conflicts of Interest** | All authors should declare all potential conflicts of interest | - | The authors declare no competing interests. |

This checklist is copyrighted by the Equator Network under the Creative Commons Attribution 3.0 Unported (CC BY 3.0) license.

1. Skrivankova VW, Richmond RC, Woolf BAR, Yarmolinsky J, Davies NM, Swanson SA, et al. Strengthening the Reporting of Observational Studies in Epidemiology using Mendelian Randomization (STROBE-MR) Statement. JAMA. 2021;under review.

2. Skrivankova VW, Richmond RC, Woolf BAR, Davies NM, Swanson SA, VanderWeele TJ, et al. Strengthening the Reporting of Observational Studies in Epidemiology using Mendelian Randomisation (STROBE-MR): Explanation and Elaboration. BMJ. 2021;375:n2233.
